# Role of Annual Influenza Vaccination against Lung Cancer in Type 2 Diabetic Patients from a Population-Based Cohort Study

**DOI:** 10.3390/jcm10153434

**Published:** 2021-08-01

**Authors:** Jing-Quan Zheng, Cheng-Hsin Lin, Chun-Chao Chen, Yuan-Feng Lin, Chun-Chih Chiu, Tsung Yeh Yang, Min-Huei Hsu, Yu-Ann Fang, Wen-Rui Hao, Ju-Chi Liu, Kang-Yun Lee

**Affiliations:** 1 Graduate Institute of Clinical Medicine, College of Medicine, Taipei Medical University, Taipei 11031, Taiwan; jingquan235@gmail.com (J.-Q.Z.); b101092035@tmu.edu.tw (C.-C.C.); d001089012@tmu.edu.tw (Y.-F.L.); b8501043@tmu.edu.tw (W.-R.H.); 2 Division of Pulmonary Medicine, Department of Internal Medicine, Shuang Ho Hospital, Taipei Medical University, New Taipei City 23561, Taiwan; 3 Division of Pulmonary Medicine, Department of Internal Medicine, School of Medicine, College of Medicine, Taipei Medical University, Taipei 11031, Taiwan; 4 Taipei Heart Institute, Taipei Medical University, Taipei 11031, Taiwan; chlin@s.tmu.edu.tw (C.-H.L.); 17257@s.tmu.edu.tw (C.-C.C.); 15535@s.tmu.edu.tw (T.Y.Y.); runawayyu@hotmail.com (Y.-A.F.); 5 Division of Cardiovascular Surgery, Department of Surgery, Shuang Ho Hospital, Taipei Medical University, New Taipei City 23561, Taiwan; 6 Division of Cardiovascular Surgery, Department of Surgery, School of Medicine, College of Medicine, Taipei Medical University, Taipei 11031, Taiwan; 7 Division of Cardiology, Department of Internal Medicine, Shuang Ho Hospital, Taipei Medical University, New Taipei City 23561, Taiwan; 8 Division of Cardiology, Department of Internal Medicine, School of Medicine, College of Medicine, Taipei Medical University, Taipei 11031, Taiwan; 9 Graduate Institute of Data Science, College of Management, Taipei Medical University, Taipei 11031, Taiwan; 01056@tmu.edu.tw; 10 Department of Neurosurgery, Wan-Fang Hospital, Taipei Medical University, Taipei 11696, Taiwan

**Keywords:** hedging, transaction costs, dynamic programming, risk management, post-decision state variable

## Abstract

Type 2 diabetes mellitus (DM) patients are at a higher risk for developing lung cancer due to immune dysfunction and chronic inflammation. They also have increased morbidity and mortality related to influenza, and it is recommended that they receive an annual influenza vaccination. In this study, we evaluate whether influenza vaccination could reduce the incidence of lung cancer in DM patients. This cohort study included DM patients (≥55 years old) between 1 January 2002 and 31 December 2012 by using the Taiwan Health Insurance Database. Cox proportional hazard regression method was used to compare the relation between the influenza vaccination and lung cancer incidence after adjusting for potential confounders. Sub-group analyses were done according to vaccination status (unvaccinated, total number of vaccinations: 1, 2–3, ≥4) and evaluated the dose-dependent effects on lung cancer events. Among 22,252 eligible DM patients, 7860 (35.32%) received an influenza vaccination and 67.68% (14392) did not receive an influenza vaccination. Lung cancer incidence was significantly lower in the vaccinated group versus the unvaccinated group (adjusted HR 0.77; 95% CI 0.62–0.95, *p* < 0.05). Significant protective effects were observed among male sex (adjusted HR 0.72; 95% CI 0.55–0.94, *p* < 0.05) and 55–64 year (adjusted HR 0.61; 95% CI 0.40–0.94, *p* < 0.05) and ≥75 year (adjusted HR 0.63; 95% CI 0.42–0.92, *p* < 0.05) age groups, respectively. A dose-dependent protective effect was noted with a significant protective effect in those that received ≥4 vaccinations (adjusted HR 0.42; 95% CI 0.29–0.61, *p* < 0.001). In sub-group analysis, elder patients with ≥65 years of age were significantly protected from ≥4 vaccinations (adjusted HR 0.37; 95% CI 0.23–0.62, *p* < 0.001 in 65–74 years and adjusted HR 0.31; 95% CI 0.15–0.66, *p* = 0.002 in ≥75 years group, respectively). Male sex with ≥4 vaccinations had a significantly lower risk of lung cancer (adjusted HR 0.35; 95% CI 0.21–0.57, *p* < 0.001). Patients with comorbid conditions that received ≥4 vaccinations were also protected, and was especially significant among those with CCI ≥ 3 (adjusted HR 0.38; 95% CI 0.18–0.80, *p* = 0.009) as compared to 1 and 2–3 vaccination groups, including those with hypertension (adjusted HR 0.35; 95% CI 0.22–0.57, *p* < 0.001). This population-based cohort study demonstrated that annual influenza vaccination significantly reduced the lung cancer risk in DM patients and specifically demonstrates that a higher number of vaccinations is related with a more protective effect. Whether this is due to vaccine booster effects on anti-tumor immune regulation among DM patients still needs to be explored.

## 1. Introduction

Taiwan’s diabetic population has increased alarmingly in recent years and its prevalence ranges between 4.9% and 9.2%. Patients with type 2 diabetes have a significant risk for a number of cancers, including liver and pancreas [1,2,3,4]. Many cohort studies revealed an elevated risk of lung cancer in diabetic patients especially among women [5,6,7]. Diabetes is regarded as an independent risk factor for lung cancer among non-smokers [8]. Preclinical studies suggested several mechanisms related to diabetes as a risk for lung cancer development and growth. Hyperinsulinemia and insulin resistance promote cancer development through stimulation of insulin-like growth factor and its receptors [9,10]. Hyperglycemia increases oxidative stress and chronic inflammation which further damage the lung structures [11]. Chronic lung damage such as emphysema is known as an independent risk factor for lung cancer development [12]. A recent meta-analysis proved that diabetes independently increased the risk of lung cancer after controlling for age, smoking and alcohol [13]. Furthermore, a meta-analysis also exhibited a relation between DM with poorer prognosis in lung cancer patients [14]. In short, lung cancer is an important and critical issue among diabetic patients.

On the other hand, since DM patients have a higher risk of developing complications with influenza infection, the Taiwan government recommends annual influenza vaccinations for diabetic patients as per the Advisory Committee on Immunization Practices (ACIP) [15]. In our previous study, we found that influenza vaccinations reduced the risk of lung cancer among chronic lung disease patients [16]. Since chronic inflammation and immune dysfunction are key shared pathogenesis of both diabetes and chronic lung destruction for lung cancer development, we consider the influenza vaccination might be of benefit to DM patients against lung cancer. Thus, in this population-based cohort study, we evaluate whether the influenza vaccination reduces lung cancer risk among DM patients.

## 2. Materials and Methods

**Figure 1:**
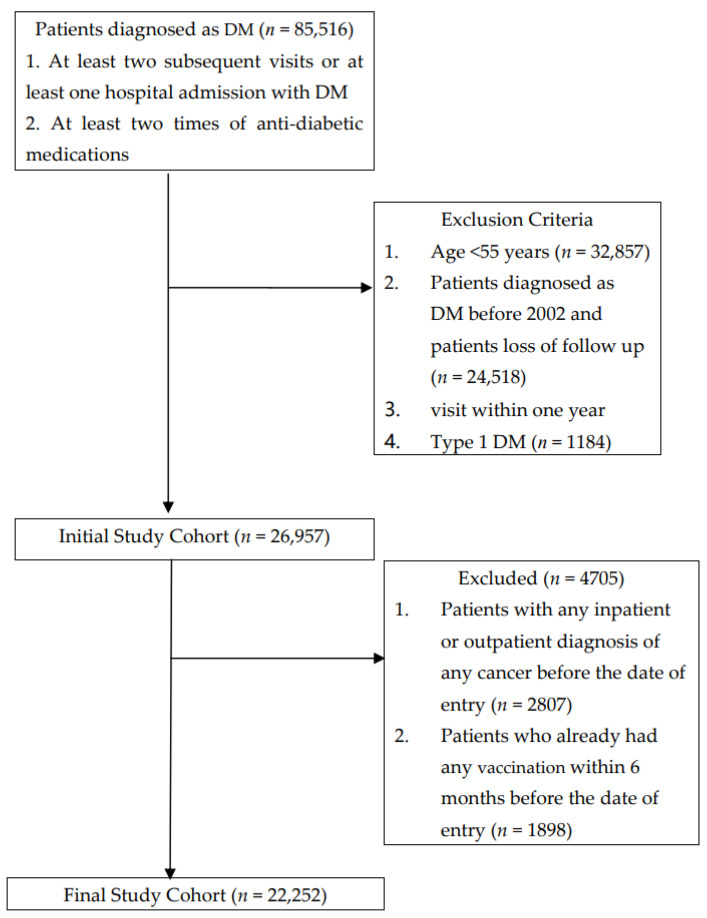
Data Selection Process.

We used the nationwide database, National Health Insurance Research Database (NHIRD) to analyze the influence of influenza vaccination on lung cancer among diabetic patients. The Taiwan National Health Insurance (NHI) program, established in 1995, is a national comprehensive health insurance provided to all residents of Taiwan and more than 98% of the total population is covered under the NHI program. We screened patients who visited all healthcare facilities with a diagnosis of type 2 diabetes mellitus (DM) (International Classification of Disease, the 9th Revision, Clinical Modification Code) over an 11 year period (*n* = 151,605) from 1 January, 2002 to 31 December, 2012. Patients with 1. at least two subsequent DM visits or one hospital admission with a diagnosis of DM and 2. at least two incidences of using diabetic medications (*n* = 85,516) were included in the study. The initial study cohort included 26,957 DM patients after exclusion of those with 1. age <55 years (*n* = 32,857), 2. diagnosed as DM before 2002 (*n* = 24,518) and 3. type I DM (*n* = 1184). The final study cohort included 22,252 DM patients after further exclusion of those 1. diagnosed with any cancer (*n* = 2807) and 2. that took any vaccination within 6 months (*n* = 1898) before the date of entry (Figure 1).

Taiwan NIH implemented mandatory influenza vaccination among the high-risk elderly population in 1998 (people aged ≥50 years with type 2 diabetes, chronic liver infection of liver cirrhosis, cardiovascular diseases, or chronic pulmonary disease, etc.) and all older people >65 years of age since 2001 [9]. The vaccination status of each study subject was recognized by code V048 and/or the use of vaccine (confirmed by drug codes). The primary endpoints of our study were incidence of lung cancer (ICD-9-CM codes 162.X) in type 2 DM patients. This study was approved by the Joint Institutional Review Board of Taipei Medical University (approval no. N201804043, on 26 April 2018). Each individual’s Charlson Comorbidity Index (CCI) and other comorbid conditions (e.g., hypertension, dyslipidemia, etc.) and the associated medications such as statins, aspirin and anti-hypertensive agents ACEI and ARB were analyzed.

### Statistical Analysis

Propensity scores (PS) method was used to reduce the selection bias in the comparison of the vaccinated group and the non-vaccinated group by accounting for the covariates with a logistic regression model [17]. The Chi-square test was used for categorical variables and the t-test was used for continuous variables. The association between influenza vaccination and lung cancer in DM patients was analyzed by Cox proportional hazards regression analysis. The influence of the dose-effect of influenza vaccinations on incidence of lung cancer was determined; DM patients were categorized into four groups according to vaccination status (unvaccinated, total number of vaccinations: 1, 2 and 3, and ≥4). These data were stratified as patients’ age, sex, comorbidity conditions, and chronic medication use. Sensitivity analysis was done to evaluate the differences and consistencies between the influenza vaccination use and the risk of lung cancer in DM patients. All statistical analyses were performed by using SPSS 22.0 and SAS 9.4 software. The significance criterion was set at *p* < 0.05.

## 3. Results

Among a total of 22,252 eligible DM patients, 7860 (35.32%) received influenza vaccinations and 67.68% (14,392) did not receive influenza vaccinations (Table 1). A significant difference (*p* < 0.001) between the two groups including distributions of age, sex, use and number of anti-diabetic medications, co-medications, level of urbanization and monthly income was observed (Table 1). The prevalence of certain preexisting medical comorbidities, including CCI index (*p* < 0.001) and hypertension (*p* < 0.001), was higher in the vaccinated than the unvaccinated group. The vaccinated group had a higher percentage of anti-diabetic medication use (including insulin and analogues, sulfonamides, alpha glucosidase inhibitors and thiazolidinediones) and of >3 anti-diabetic medications than the unvaccinated group (*p* < 0.001). Analyzing comorbidities-associated medication use revealed a longer use of statins (*p* < 0.001), aspirin (*p* < 0.001), anti-hypertensive agent, angiotensin-converting enzyme (ACE) inhibitor and angiotensin receptor blocker (ARB) (*p* < 0.001) among the vaccinated group than in the unvaccinated group.

We analyzed the incidence of lung cancer among DM patients with or without influenza vaccination (Table 2). After adjustment for potential confounders, the incidence of lung cancer was significantly lower in the vaccinated group as compared to the unvaccinated group (adjusted HR 0.77; 95% CI 0.62–0.95, *p* < 0.05). Significant protective effects were observed among the male sex (adjusted HR 0.72; 95% CI 0.55–0.94, *p* < 0.05) and the 55–64 years (adjusted HR 0.61; 95% CI 0.40–0.94, *p* < 0.05) and ≥75 years (adjusted HR 0.63; 95% CI 0.42–0.92, *p* < 0.05) age groups, respectively (Table 2).

The sensitivity analysis adjustments were done to evaluate the association of influenza vaccinated patients with the risk of lung cancer in different models, stratifying by the total number of vaccinations (Table 3). Interestingly, a dose-dependent protective effect was noted in the main model with a significant protective effect in those that received ≥4 vaccinations (adjusted HR 0.42; 95% CI 0.29–0.61, *p* < 0.001). In sub-group analysis, elder patients over ≥65 years of age were significantly protected from ≥4 vaccinations (adjusted HR 0.37; 95% CI 0.23–0.62, *p* < 0.001 in 65–74 years and adjusted HR 0.31; 95% CI 0.15–0.66, *p* = 0.002 in ≥75 years group, respectively). Male sex with ≥4 vaccinations had a significantly lower risk for lung cancer (adjusted HR 0.35; 95% CI 0.21–0.57, *p* < 0.001). Patients with ≥4 vaccinations were also protected among those with comorbid conditions, especially significant among those with CCI ≥3 (adjusted HR 0.38; 95% CI 0.18–0.80, *p* = 0.009) as compared to 1 and 2–3 vaccination groups, including those with hypertension (adjusted HR 0.35; 95% CI 0.22–0.57, *p* < 0.001). However, the protective effects of vaccination doses did not significantly differ with type or number of anti-diabetic medications (Table 3).

## 4. Discussion

Our DM population revealed that patients in the vaccinated group were older, female prevalent, had higher CCI index scores and related with hypertension more than the unvaccinated group. Significantly more patients in the vaccinated group had more anti-diabetic agents use and longer co-medications use as compared to the unvaccinated group. As per Taiwan government-funded influenza vaccinations policy, all diabetic patients are considered to be at high risk from influenza infection and mortality, and were recommended to take the vaccination [15]. To our knowledge, no previous study has been reported about the influence of influenza vaccinations on lung cancer risk among diabetic patients. This is the first population-based cohort study that revealed the significant reduction of lung cancer risk with influenza vaccination among type 2 DM patients. An impairment of metabolism-immune axis in type 2 DM is believed to relate with development and progression of neoplasm [18,19]. Reduced glycolysis and basal cellular mitochondrial respiration in type 2 diabetes mellitus was related with decreased circulating CD8 + T cells and led to the production impairment of various cytokines [20]. Most of the identified studies state that the immunogenicity of influenza vaccination among DM patients is comparable to healthy or non-diabetic patients [21,22,23]. The seasonal influenza vaccination generates systemic CD8+ T cell-mediated antitumor immunity [24], which also boosts the response to anti-tumor treatment.

After adjustments for age, sex, CCI Index, co-morbid conditions, medications use, level of urbanization and monthly income, the lung cancer incidence was significantly lower in the influenza vaccinated group. This trend was reduced in both sexes, however, it was more significant in diabetic men. The higher prevalence of smoking among male patients might explain the significant lung cancer prevention from influenza vaccination over female patients. In Taiwan, the smoking rates for 55 years or older males and females were 20.5% and 2.4% in 2002; 14% and 1.53% in 2012, respectively [25]. The lower smoking rate in females might be related with traditional Chinese culture. Tobacco use has been reported to be the main cause of lung cancers and the risk is 20–40 times higher in smokers than non-smokers [26]. Through damaging the local cellular and humoral immunity in respiratory system [27,28], smoking facilitates influenza viral infection and consequent pneumonia [29,30]. A previous study also revealed that smokers and ex-smokers had an increased risk of influenza related hospitalization and severity [31]. Other studies also revealed a sex difference in antibody response after influenza vaccination [32,33,34]. However, the mechanism underlying such lung cancer protective effects of influenza vaccination on gender difference is not fully understood.

Interestingly, the potential “dose-dependent” protective effect was noted between cumulative doses of influenza shot and the risk of lung cancer among type 2 DM patients. Further analysis revealed that patients of increasing age ≥65 years, regardless of sex and CCI index, those with hypertension and those with fewer anti-diabetic medications were significantly protected by ≥4 vaccination. Higher vaccination dose requirements in elder male patients could be related with their smoking status, since smoking reduced the effectiveness of the influenza vaccination [31]. We couldn’t determine whether the patients received annual vaccinations yearly for more than 4 years or received vaccinations ≥ 4 times over the observation period. However, from our study, its protective effect seemed to be related to the cumulative dose effect. A previous study in the Taiwanese population revealed that cumulative exposure to influenza infection increased the risk of lung cancer [35]. The study revealed a 25% increased risk of lung cancer in patients with ≥5 episodes of influenza infection [35]. This might explain our finding of cumulative anti-influenza shots being related to decreased risk for lung cancer, and the underlying mechanisms might be due to improvements in chronic inflammation and booster immune status against lung cancer among DM patients. Future in vitro and in vivo studies are needed to investigate these underlying mechanisms and broaden the knowledge of influenza vaccination to prevent and treat lung cancer.

This is a population-based study on type 2 DM patients regarding the possible protective effects of influenza vaccine against lung cancer. Several limitations still need to be considered as with other observational studies based on clinical databases. Important environmental risk factors for lung cancer such as smoking status was not available from this database. No cause–relation could derive from such a database, however, we revealed a significant reduction of lung cancer risk in male patients. Since a previous meta-analysis study revealed that diabetes mellitus itself is related to an increased risk for lung cancer after adjusting for smoking status [13], we suppose the use of vaccination in male diabetic patients is still important. Second, the diagnoses of diabetes mellitus, comorbidities and medication use were identified based on the ICD-9 codes or drug codes alone. We used propensity score (PS) matching to reduce the selection bias by accounting for the covariates with a logistic regression mode [17]. We further applied sensitivity analyses and adjustments of confounding factors using a large number of stratifications to compare between the groups to avoid the influence of interference factors. Moreover, as an intrinsic weakness of the database, no biochemical data was available and we couldn’t evaluate the duration or severity of DM in our population. Other unmeasured confounders, including body mass index, alcohol drinking, and use of over-the-counter medications, etc., were not available. We also couldn’t identify which brand of vaccine our population received from such a database; however, in Taiwan, patients over 55 years with diabetes mellitus were eligible and recommended for publicly funded influenza vaccine. Since qualified people received flu shots at public expense free of charge, most of them took similar brands of influenza vaccine. Though we couldn’t detect those without public brands, we believe the number was negligible and might not influence our results. Although these limitations did not seem to compromise the significant findings of our results, we still can’t establish a cause–effect relationship from our study; we still need future prospective randomized studies to verify our findings.

## 5. Conclusions

Our study demonstrated the importance of administering influenza vaccinations among diabetic patients for preventing lung cancer. We recommend influenza vaccination in all diabetic patients regardless of age, sex and co-morbid conditions and specifically believe that these patients might need more influenza vaccinations to boost its protective effects against lung cancer. Why and how cumulative doses of influenza vaccinations should be enough for these population is still unclear and needs to be explored through future prospective trials.

## Figures and Tables

**Table 1:** Characteristics of the Study Population.

	Whole Cohort (*n* = 22,252)		Unvaccinated (*n* = 14,392)		Vaccinated (*n* = 7860)		*p*
	*n*	%	*n*	%	*n*	%	
Age, years (Mean ± SD)	66.48 (8.70)		64.64 (8.74)		69.85 (7.54)		<0.001
55–64	11,464	51.52	9111	63.31	2353	29.94	<0.001
65–74	6749	30.33	3202	22.25	3547	45.13	
≥75	4039	18.15	2079	14.45	1960	24.94	
Gender							
Female	11,248	50.55	7032	48.86	4216	53.64	<0.001
Male	11,004	49.45	7360	51.14	3644	46.36	
CCI Index							
0	6821	30.65	4743	32.96	2078	26.44	<0.001
1	5959	26.78	3881	26.97	2078	26.44	
2	4322	19.42	2689	18.68	1633	20.78	
≥3	5150	23.14	3079	21.39	2071	26.35	
Hypertension							
No	7775	34.94	5389	37.44	2386	30.36	<0.001
Yes	14,477	65.06	9003	62.56	5474	69.64	
Dyslipidemia							
No	14,300	64.26	9263	64.36	5037	64.08	0.679
Yes	7952	35.74	5129	35.64	2823	35.92	
Hypoglycemic medications							
Insulin and analogues	3788	17.02	2242	15.58	1546	19.67	<0.001
Biguanides	16,711	75.10	10,813	75.13	5898	75.04	0.877
Sulfonamides, urea derivatives	14,330	64.40	8980	62.40	5350	68.07	<0.001
Alpha glucosidase inhibitors	3889	17.48	2325	16.15	1564	19.90	<0.001
Thiazolidinediones	2949	13.25	1770	12.30	1179	15.00	<0.001
Dipeptidyl peptidase 4 (DPP-4)	3089	13.88	1975	13.72	1114	14.17	0.353
Oral blood glucose lowering	2808	12.62	1843	12.81	965	12.28	0.257
Other blood glucose lowering drugs	2917	13.11	1700	11.81	1217	15.48	<0.001
Number of hypoglycemic medications							
0–1	6652	29.89	4522	31.42	2130	27.10	<0.001
2-3	9987	44.88	6587	45.77	3400	43.26	
>3	5613	25.22	3283	22.81	2330	29.64	
Combined medications							
Statins							
<28 days	11,051	49.66	7428	51.61	3623	46.09	<0.001
28–365 days	5150	23.14	3418	23.75	1732	22.04	
>365 days	6051	27.19	3546	24.64	2505	31.87	
Aspirin							
<28 days	12,296	55.26	8908	61.90	3388	43.10	<0.001
28–365 days	4192	18.84	2535	17.61	1657	21.08	
>365 days	5764	25.90	2949	20.49	2815	35.81	
ACEI and ARB							
<28 days	8138	36.57	6026	41.87	2112	26.87	<0.001
28–365 days	4765	21.41	3174	22.05	1591	20.24	
>365 days	9349	42.01	5192	36.08	4157	52.89	
Level of Urbanization							
Urban	15,587	70.05	10,619	73.78	4968	63.21	<0.001
Suburban	4432	19.92	2626	18.25	1806	22.98	
Rural	2233	10.04	1147	7.97	1086	13.82	
Monthly income (NT$)							
0	2016	9.06	1129	7.84	887	11.28	<0.001
1–19,200	6212	27.92	3687	25.62	2525	32.12	
19,200–25,000	6746	30.32	3896	27.07	2850	36.26	
≥25,001	7278	32.71	5680	39.47	1598	20.33	

CCI Index, Charlson Comorbidity Index. ACEI, angiotensin converting enzyme inhibitor. ARB, angiotensin II receptor blocker.

**Table 2:** Risk of Lung Cancer among Unvaccinated and Vaccinated DM patients.

All Group (*n* = 22,252)	Unvaccinated (Total Follow-Up 59,866.2 Person-Years)		Vaccinated (Total Follow-Up 51,033.7 Person-Years)		Adjusted HR † (95% C.I.)
	No. of Patients With Cancer	Incidence Rate (per 10^5^ Person-Years) (95% C.I.)	No. of Patients With Cancer	Incidence Rate (per 10^5^ Person-Years) (95% C.I.)	
Whole cohort	244	407.6 (356.4, 458.7)	165	323.3 (274.0, 372.6)	0.77 (0.62, 0.95) *
Age (years)					
55–64 ^a^	112	268.9 (219.1, 318.7)	28	151.9 (95.6, 208.2)	0.61 (0.40, 0.94) *
65–74 ^b^	74	600.0 (463.3, 736.8)	87	386.1 (305.0, 467.3)	0.74 (0.54, 1.03)
≥75 ^c^	58	986.4 (732.5, 1240.2)	50	496.5 (358.9, 634.1)	0.63 (0.42, 0.92) *
Sex					
Female ^d^	77	254.3 (197.5, 311.1)	64	226.4 (170.9, 281.8)	0.85 (0.60, 1.22)
Male ^e^	167	564.4 (478.8, 650.0)	101	443.7 (357.2, 530.2)	0.72 (0.55, 0.94) *

*: *p* < 0.05. ^a^ Total follow-up 41,653.7 person-years for unvaccinated and 18,432.9 for vaccinated. ^b^ Total follow-up 12,332.4 person-years for unvaccinated and 22,530.3 for vaccinated. ^c^ Total follow-up 5880.1 person-years for unvaccinated and 10,070.5 for vaccinated. ^d^ Total follow-up 30,276.8 person-years for unvaccinated and 28,270.6 for vaccinated. ^e^ Total follow-up 29,589.4 person-years for unvaccinated and 22,763.1 for vaccinated. C.I.: confidence interval. HR: hazard ratio. † Main model is adjusted for age, sex, CCI Index, Hypertension, Dyslipidemia, INSULINS AND ANALOGUES, Biguanides, Sulfonamides, urea derivatives, Alpha glucosidase inhibitors, Thiazolidinediones, Dipeptidyl peptidase 4, oral blood glucose lowering, other blood glucose lowering drugs, anti-diabetes medications, statins, Aspirin, ACEI and ARB, level of urbanization, monthly income in propensity score.

**Table 3:** Sensitivity Analysis of Adjusted HRs of Vaccination in Risk Reduction of Lung Cancer among DM patients.

	Unvaccinated	Vaccinated			*p* for Trend
		1	2–3	≥4	
	Adjusted HR (95% C.I.)	Adjusted HR (95% C.I.)	Adjusted HR (95% C.I.)	Adjusted HR (95% C.I.)	
Main model †	1.00	0.95(0.72, 1.26)	0.88(0.67, 1.16)	0.42(0.29, 0.61) ***	<0.001
Subgroup effects					
Age, years					
55–64	1.00	0.60(0.32, 1.11)	0.66(0.35, 1.24)	0.56(0.24, 1.28)	0.041
65–74	1.00	1.08(0.71, 1.64)	0.90(0.60, 1.36)	0.37(0.23, 0.62) ***	<0.001
≥75	1.00	0.82(0.49, 1.37)	0.68(0.41, 1.12)	0.31(0.15, 0.66) **	0.002
Sex					
Female	1.00	1.01(0.63, 1.63)	0.97(0.61, 1.53)	0.53(0.30, 0.95) *	0.076
Male	1.00	0.93(0.65, 1.32)	0.84(0.59, 1.20)	0.35(0.21, 0.57) ***	<0.001
CCI Index					
0	1.00	0.97(0.58, 1.62)	0.72(0.41, 1.26)	0.35(0.17, 0.75) **	0.006
1	1.00	0.71(0.37, 1.34)	1.08(0.63, 1.86)	0.50(0.25, 0.99) *	0.130
2	1.00	1.20(0.66, 2.17)	1.03(0.57, 1.86)	0.40(0.16, 0.97) *	0.124
≥3	1.00	0.89(0.52, 1.52)	0.70(0.40, 1.21)	0.38(0.18, 0.80) *	0.009
Hypertension					
No	1.00	1.11(0.70, 1.76)	1.14(0.72, 1.81)	0.56(0.31, 1.01)	0.196
Yes	1.00	0.86(0.60, 1.24)	0.77(0.54, 1.09)	0.35(0.22, 0.57) ***	<0.001
Dyslipidemia					
No	1.00	0.90(0.63, 1.27)	0.98(0.70, 1.36)	0.39(0.24, 0.62) ***	0.001
Yes	1.00	1.06(0.65, 1.72)	0.69(0.41, 1.16)	0.46(0.24, 0.85) *	0.010
Insulins and analogues					
No (<28 days)	1.00	0.93(0.69, 1.26)	0.84(0.62, 1.13)	0.36(0.23, 0.54) ***	<0.001
Yes (≥28 days)	1.00	1.07(0.49, 2.33)	1.12(0.54, 2.34)	0.75(0.32, 1.75)	0.666
Biguanides					
No (<28 days)	1.00	0.73(0.47, 1.13)	0.67(0.43, 1.04)	0.30(0.15, 0.59) ***	<0.001
Yes (≥28 days)	1.00	1.09(0.75, 1.58)	1.02(0.71, 1.46)	0.49(0.31, 0.77) **	0.013
Sulfonamides, urea derivatives					
No (<28 days)	1.00	0.81(0.53, 1.25)	0.59(0.37, 0.94) *	0.35(0.19, 0.65) ***	<0.001
Yes (≥28 days)	1.00	1.04(0.72, 1.52)	1.08(0.76, 1.54)	0.45(0.28, 0.72) ***	0.011
Alpha glucosidase inhibitors					
No (<28 days)	1.00	0.86(0.63, 1.17)	0.85(0.63, 1.15)	0.41(0.27, 0.62) ***	<0.001
Yes (≥28 days)	1.00	1.63(0.78, 3.40)	1.04(0.47, 2.32)	0.45(0.16, 1.24)	0.197
Thiazolidinediones					
No (<28 days)	1.00	0.94(0.70, 1.26)	0.81(0.60, 1.09)	0.37(0.25, 0.55) ***	<0.001
Yes (≥28 days)	1.00	0.96(0.35, 2.60)	1.57(0.69, 3.62)	0.88(0.31, 2.54)	0.783
Dipeptidyl peptidase 4 (DPP-4)					
No (<28 days)	1.00	0.93(0.70, 1.24)	0.85(0.64, 1.12)	0.40(0.28, 0.59) ***	<0.001
Yes (≥28 days)	1.00	0.61(0.07, 5.21)	1.50(0.35, 6.34)	0.46(0.05, 4.30)	0.782
Oral blood glucose lowering					
No (< 28 days)	1.00	0.98(0.73, 1.31)	0.88(0.66, 1.18)	0.43(0.29, 0.63) ***	<0.001
Yes (≥28 days)	1.00	0.44(0.10, 1.96)	0.72(0.22, 2.31)	0.14(0.02, 1.15)	0.062
Other hypoglycemic medications					
No (<28 days)	1.00	0.99(0.74, 1.32)	0.80(0.59, 1.08)	0.39(0.26, 0.59) ***	<0.001
Yes (≥28 days)	1.00	0.38(0.09, 1.66)	1.66(0.75, 3.65)	0.54(0.17, 1.69)	0.822
Number of hypoglycemic medications					
0–1	1.00	0.70(0.45, 1.10)	0.55(0.34, 0.87) *	0.32(0.17, 0.59) ***	<0.001
2–3	1.00	1.34(0.89, 2.03)	1.02(0.66, 1.57)	0.42(0.23, 0.76) **	0.022
>3	1.00	0.66(0.29, 1.51)	1.30(0.69, 2.44)	0.53(0.23, 1.21)	0.387

*: *p* < 0.05; **: *p* < 0.01; ***: *p* < 0.001; HR: hazard ratio; † Main model is adjusted for age, sex, CCI Index, Hypertension, Dyslipidemia, Insulins and analogues, Biguanides, Sulfonamides, urea derivatives, Alpha glucosidase inhibitors, Thiazolidinediones, Dipeptidyl peptidase 4, oral blood glucose lowering, other blood glucose lowering drugs, anti-diabetes medications, Statins, Aspirin, ACEI and ARB, level of urbanization, monthly income in propensity score.
